# USACE Coastal and Hydraulics Laboratory Quality Controlled, Consistent Measurement Archive

**DOI:** 10.1038/s41597-022-01344-z

**Published:** 2022-05-30

**Authors:** Candice Hall, Robert E. Jensen

**Affiliations:** 1 USACE Engineer Research and Development Center, Vicksburg, MS USA; 2 Department of Oceanography, University of Cape Town, Cape Town, South Africa

**Keywords:** Physical oceanography, Physical oceanography, Water resources, Natural hazards, Environmental monitoring

## Abstract

The US Army Corps of Engineers (USACE) utilizes the National Oceanic and Atmospheric Administration (NOAA) National Data Buoy Center (NDBC) buoy measurements for validation of their wave models and within coastal applications. However, NDBC data are accessible via multiple archives; each with their own source-specific storage, metadata, and quality control protocols, which result in inconsistencies in the accessible data. Therefore, USACE has developed an independent, quality controlled, consistent (QCC) Measurement Archive that captures the best available NDBC observations with verified metadata. This work details the methodology behind this USACE QCC Measurement Archive; showcasing improvements in data quality via geographical location and wave parameter examples. Note that this methodology only removes known erroneous data, it does not verify data quality from an alternate source. This self-describing, USACE QCC Measurement Archive therefore provides a database of consistently stored, geographically QA/QC’d NDBC data and metadata.

## Abstract (summary)

**Table:** 

Measurement(s)	atmospheric wind speed • atmospheric wind direction • wind gust • pressure of air • temperature of air • temperature of sea surface • significant wave height • wave period • wave direction • wave spectral energy • wave spectral direction
Technology Type(s)	anemometer • barometer • Thermometer Device • wave sensor
Sample Characteristic - Organism	none
Sample Characteristic - Environment	sea state • ocean • temperature of sea surface • sea water environment • coastal sea water • sea water • ocean time series station • marine observatory system • marine environment
Sample Characteristic - Location	United States of America

## Background & Summary

One of the U.S. Army Corps of Engineers (USACE) missions is to oversee operations and maintenance activities in the coastal waters of the U.S. These activities include sediment transport, hardened structures, harbor navigability, climate resilience and coastal protection, all of which require knowledge and assessment of the wave climate. For practical assessments, USACE wave related technologies require accurate and homogeneous wave measurements from *in situ* observational platforms.

To that end, USACE sponsored an investigation into uncertainty errors in the wave measurement systems that are used for evaluating products such as their Wave Information Study (WIS), a wave hindcast effort that serves as the basis for resolving the U.S. wave climate. Of particular interest are measurement errors that may compromise wave model evaluations. These errors may be indistinguishable from wind forcing or wave model deficiencies, and may transfer into other USACE wave and coastal applications.

One source of validation data is the National Oceanic and Atmospheric Administration (NOAA) National Data Buoy Center (NDBC) *in situ* buoy meteorological and wave measurements. As of 2022, NDBC publishes their data via two different streams: real time and historical. The real time data feed undergoes broad, automated QA/QC protocols^1^ to meet emergency management and forecasting agency latency commitments that require swift publication to the Global Telecommunications System (GTS). These ‘Real Time Data’ files are also published within individual stations pages on the NDBC website as tabular files that are continually updated and cover the last forty-five days (e.g. https://www.ndbc.noaa.gov/station_realtime.php?station = 41009).

Once latency commitments are met, NDBC manually QA/QC’s^1^ these data and stores them within station specific ‘Historical Data’ text files on their website on a monthly basis (e.g. https://www.ndbc.noaa.gov/station_history.php?station = 41009). As per NOAA requirements, NDBC archives their data on a monthly basis in the official NOAA archives, which are found at the National Center for Environmental Information (NCEI; https://www.ncei.noaa.gov/access/marine-environmental-buoy-database/). NDBC collates their website data annually and copy these data, in a Unidata’s Network Common Data Form (netCDF) format, for storage on the NDBC Distributed Oceanographic Data Systems framework (DODS; https://dods.ndbc.noaa.gov/thredds/catalog/data/catalog.html). Essentially, the NDBC website and the DODS may be considered as a single source of NDBC historical data that are stored in different formats.

Over the decades, the NDBC data have experienced technological advances in instrumentation and archival storage. While NDBC has invested in minimizing instrumentation effects on its datasets^2–4^, these various archives present their own set of specific storage procedures that influence data quality. These influences augment measurement discontinuities that are detectable within observation wave time series data^5–9^, which were previously attributed to instrumentation or platform exchanges.

Therefore USACE undertook a thorough examination of the differences between these two NDBC and official NOAA archives at NCEI resources. The detected differences are detailed within a USACE Coastal and Hydraulics Engineering Technical Note^10^. In essence, the published NDBC website data, sourced from the in-house NDBC database that are subjected to manual NDBC QA/QC procedures^1^, presents a consistent, uniform formatting of historical variables and nomenclature. However the NDBC website data do not contain any metadata (geographical coordinates, instrumentation or software versioning information) that provide context to collection conditions. The official NOAA archive at NCEI, stored in netCDF format on the NCEI server, does contain those station, buoy and instrument metadata.

Unfortunately, since the implementation of the netCDF archiving process at NDBC in 2011, the NDBC data that are sourced for storage at NCEI are extracted from the automatically QA/QC’d NDBC Real Time Data stream. The automated NDBC QA/QC process flags suspect data but only removes communication transmission errors and total sensor failures^1^. Suspect data flagged during automated NDBC QA/QC require manual inspection before data removal^1^. Therefore data quality issues not detected by the automated NDBC QA/QC protocols (such as gust wind values where wind speed and direction are not available, as well as directional data exceeding 360°), including errors introduced by the NDBC netCDF data file construction process (such as duplicated data that are 5–10 seconds apart and erroneous wave frequency bands), are retained within the official NOAA archives of NDBC data at NCEI.

Due to USACE’s data consistency and metadata requirements, these NDBC archive errors^10^ necessitated the development of an in-house USACE procedure that combined data from these archives to retain the manually QA/QC’d data and attached the required metadata. This procedure developed a best available, quality controlled and consistent measurement archive (herewith called the USACE QCC Measurement Archive) with accurately described metadata. The self-describing USACE QCC Measurement Archive is actively updated on an annual basis and stored on the USACE Coastal and Hydraulic Laboratory (CHL) Data server (https://chldata.erdc.dren.mil/), accessible to both the USACE and the public. It is the methodology that created the NDBC portion of this USACE QCC Measurement Archive that is described in this manuscript.

## Methods

As NDBC publish their historical and real time *in situ* wave and meteorological data in multiple online locations, USACE developed a methodology to combine these data sources and develop a unique USACE QCC Measurement Archive that is fully self-describing. This required merging the manually quality controlled data that is stored on the NDBC website with the lower quality netCDF data with metadata files for the same stations that are stored at NCEI. The NOAA DODS source was not included as those data are exact copies of what is found within the NDBC historical station pages.

As mentioned, the NDBC website historical station pages contain the cleanest data that has been subjected to manual QA/QC by NDBC Mission Control data analysts. Data collected during service periods (when the buoys were physically on board ships for maintenance) were removed during the manual QA/QC, and are typically not present within the NDBC website data. However this data source contains no metadata other than date and time. This lack of metadata allows for the erroneous inclusion of unidentifiable data from historical time periods where the moored buoys were adrift (inaccurate wave readings, wind, temperature etc.). Additionally, although NDBC switched to a redundant meteorological sensor paradigm during the last decade, only single variable values are available per time stamp per station on the NDBC website. This is because NDBC toggles the release of primary and secondary sensor data to ensure that the highest quality data are published. However, the NDBC website contains no associated metadata indicating when these data release switches occur and hence instrumentation usage is indeterminable. Users often need these sensor details, for example wind sensor height above sea level to extrapolate wind speed at additional heights above the moored buoy. The NDBC website also does not store uncorrected non-directional spectral energy estimates (${c}_{11}^{m}$).

Conversely, the NDBC netCDF data stored at NCEI includes metadata such as time-stamped GPS positions, instrumentation metadata, data quality flags^1^, and data release flags (indicating which data were released to the real time stream). These GPS positions allow for the identification of data that was collected while NDBC moored buoys were adrift. For ease of data source identification, these NDBC netCDF files stored at NCEI will be referred to as NCEI netCDF data below. However, readers should remember that these are all NDBC data, with time-paired values that are collected from the same, unique sensor.

This NCEI netCDF data source also includes both the primary and secondary redundant meteorological sensor outputs, with metadata, as well as uncorrected non-directional spectral energy estimates (${c}_{11}^{m}$). These primary and secondary sensor variables are only found within these NCEI netCDF datasets. However, since 2011, these netCDF data are pulled from the NDBC real-time data stream, which is only subjected to automated QA/QC protocols that flag but do not remove suspect data^1^. Prior to 2011, the NDBC data were stored in an encoded Trusted Data Format (TDF), but these data were converted into netCDF format in early 2020.

Of note is that the NCEI netCDF structures differ for data stored before and after the 2011 switch to netCDF file usage. Throughout the historical netCDF dataset, the netCDF file structures contain non-uniform netCDF formats that are dependent on the data collected during file-specific time periods. Additionally, the pre-2011 netCDF files contain a nominal, fixed deployment position that is repeated for each date/time stamp within the datasets. Furthermore, these pre-2011 netCDF files contain erroneous spectral wave frequency bands that are not included in the NDBC website datasets (and do not match any wave instrumentation frequencies that NDBC has historically deployed). Both formats include instrumentation metadata that are not only inconsistent throughout the years, but within individual netCDF file’s group attributes.

**Fig. 1:**
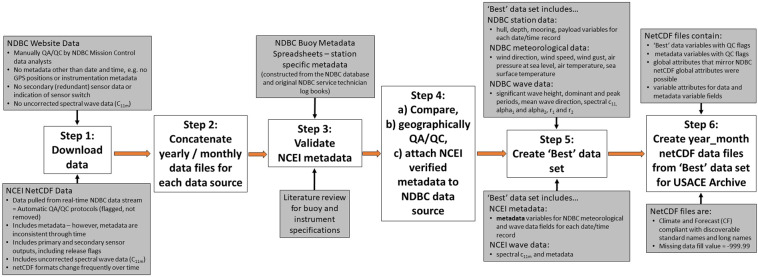
Flowchart of the USACE QCC Measurement Archive methodology. This flowchart outlines input data sources, station and metadata verification, selected ‘best’ data sets and output netCDF files.

Therefore, to mitigate these identified data source issues^10^, the USACE QCC Measurement Archive process utilized a methodology (Fig. 1) that combines each dataset’s advantages to develop a best available historical NDBC measurement dataset. For example, the GPS data included within the post-2011 NCEI netCDF files were used to detect data that fell outside a reasonable radius of the moored buoy. Conversely, the NDBC website data were used to isolate which primary or secondary sensor data were released to the public – achieved by matching the individual NDBC variable values to the equivalent primary or secondary NCEI netCDF values, therefore identifying the correct netCDF metadata. Additional outlier QA/QC variable checks, station and metadata verification (provided by literature reviews and historical NDBC buoy deployment log books) allowed for the development of a best available, self-described USACE QCC Measurement Archive.

The USACE QCC Measurement Archive methodology process consists of two phases. The first phase of the project processes the historical data, while a second phase annually appends newly available data to the historical database. The data archive routine involves a six step process (Fig. 1) for each buoy station: (1) download, (2) concatenation, (3) metadata verification, (4) comparison, geographical QA/QC and metadata attachment, (5) best dataset selection, and (6) netCDF data file creation. Finally these netCDF files are uploaded to the buoy section of the USACE CHL Data server.

These steps were automated using scripts developed in R software^11^. Where necessary, each script was subset to handle the particular idiosyncrasies^10^ of the NDBC and NCEI netCDF data archives. To process all of the historical NDBC data (1970–2021), steps two to five in phase one required ~ 400k cpu hours at the Department of Defense (DOD) Supercomputing Resource Center.

The following steps outline the methodologies utilized within this USACE QCC Measurement Archive development. For more detailed information, please see the USACE QCC Measurement Archive Standard Operating Procedure (SOP) document that is stored in the Archive GitHub (https://github.com/CandiceH-CHL/USACE_QCC_measurement_archive.git).

**Fig. 2:**
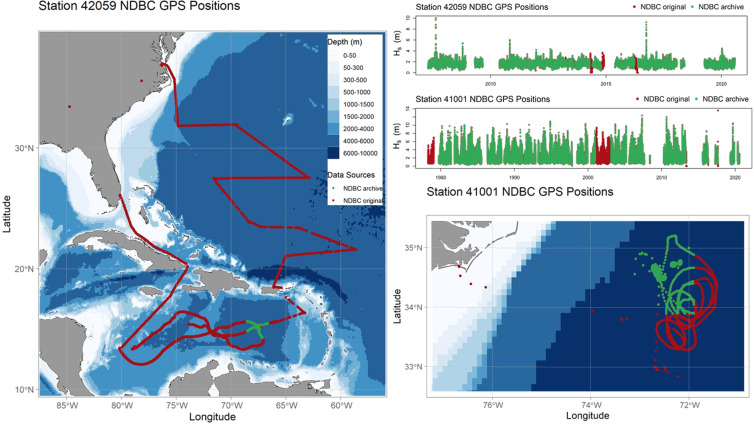
NDBC Stations 42059 (left map) and 41001 (bottom right map) buoy locations before and after geographically QA/QC of the GPS data. Map plots showcase the buoy movement, while time series plots of significant wave height (m) showcase the data collected while the buoys were adrift. In all plots, green points and track lines represent data within a one degree radius of the deployment location, while red points and track lines are outside of that range.

1. Step 1: **Download**. Historical NDBC data for all NDBC stations are downloaded from the NDBC website and the NCEI archives. Source-specific archive download links are listed in the USACE QCC Measurement Archive SOP. Data from the storage specific files types (detailed below) are extracted for concatenation in step 2.
  The NDBC website stores data in zipped yearly and monthly files as standard meteorological (stdmet), spectral wave density (swden), spectral wave (alpha_1_) direction (swdir), spectral wave (alpha_2_) direction (swdir2), spectral wave (r_1_) direction (swr1), and spectral wave (r_2_) direction data (swr2). These files require unzipping. Included within the NDBC stdmet datasets are collected meteorological and bulk wave data in the following structure: wind direction (°), wind speed (m/s), wind gusts (m/s), significant wave height (m), dominant wave period (seconds), average wave period (seconds), mean wave direction (°), air pressure at sea level (hPa), air temperature (°C), water temperature (°C), dew point temperature (°C), visibility (miles) and tide (ft). Visibility and tide are no longer collected by NDBC, and are disregarded.
  The NCEI website stores monthly NDBC files per year in netCDF format. All available data and metadata are extracted from these netCDF files. These files contain the same NDBC data as listed above, but also include additional wave spectral parameters such as uncorrected spectral energy wave data (${c}_{11}^{m}$), spectral wave co- and quad-spectra, and four wave data quality assurance parameters that are produced by the NDBC wave processing procedure^12^.
  The NCEI netCDF file formats differ significantly before and after January 2011. After January 2011, these netCDF structures varied throughout the years as NDBC buoy structures and netCDF creation procedures changed. Each format requires format-specific code to extract the data from the variable fields.
  For example, the pre-2011 netCDF files consistently contain all variables directly within the main file directory. However, the post-2011 netCDF files are structured by ‘payload’, with subset sensor fields (e.g. ‘anemomenter_1’), which in turn have their own subset variable fields (e.g. wind_speed, ‘wind_direction’) with associated quality control and release flags. Therefore users have to navigate through the payload and sensor subfields to discover the variable data with their associated metadata.
  Importantly, these ‘payload’ fields do not always refer to the on-board computer system that serves the sensor suites, e.g. NDBC’s Automated Reporting Environmental System^13^ (ARES), but also delineate between sensor suites with available primary and secondary sensor data (e.g. ‘payload_1’, ‘payload_2’). Conversely these primary and secondary sensor data (e.g. ‘air_temperature_1’ and ‘air_temperature_2’) may be subset within a single ‘payload’. Of note is that these multiple payloads often contain duplicated data.
  These ‘payload’ fields are also important when extracting data captured by NDBC Self-Contained Ocean Observations Payloads (SCOOP), as these netCDF files resemble the physical structure of the buoy stations with their modular sensor assembly. For example, the NCEI netCDF July 2020 data file for station 41009 includes 5 payload subsections. ‘payload_1’ contains an ‘anemometer_1’ sensor suite, which contains subset wind variables and data flags; ‘barometer_1’, with subset air pressure variables and flags; and a ‘gps_1’ sensor suites, with subset lat, lon variables, etc. ‘payload_2’ contains a second ‘anemometer_1’, ‘barometer_1’, ‘gps_1’, ‘air_temperature_sensor_1’, and ‘humidity_sensor_1’ suites. Payload 3 contains a single ‘gps_1’ fields (lat and lon variables with flags), while payloads 4 and 5 house ‘wave_sensor_1’ and ‘ocean_temperature_sensor_1’ sensor suites respectively, both with their own ‘gps_1’ data. In this example, ‘payload_1’ represents an R.M. Young sensor, while ‘payload_2’ is listed as a MetPak Weather Station instrument in the netCDF sensor suite attributes.
  NDBC is in the process of redesigning these netCDF file formats to be more user friendly. However, they do not plan to reformat their archive datasets. For more details on the NDBC and NCEI netCDF file formats and code extraction descriptions, please see the USACE QCC Measurement Archive SOP within the Archive GitHub.
2. Step 2: **Concatenation**. This step merges each yearly and monthly data files to produce a single time series of concatenated stdmet data, and time series files for each individual spectral wave variable. The concatenated stdmet data format mirrors the NDBC website data formats. To handle the NDBC data, this step allows for the management of differing yearly file formats and spectral frequencies; the concatenation of multiple date and time columns into one field; and the removal of redundant date, time and tide columns in stdmet data. This step allocates the spectral data into the standard NDBC 38 frequencies (old wave sensors), and 47 frequencies (new wave sensors). Finally, this step converts the NDBC r_1_ and r_2_ values to their correct units (NDBC r_1_ and r_2_ data are scaled by 100 to reduce storage requirements, so these data should be multiplied by 0.01).
  To handle the NCEI data, this step allows for the concatenation of stdmet data to create a dataset that matches the NDBC website data nomenclature. This step also removes data that were flagged as erroneous by automated NDBC QA/QC protocols. As unit standards vary between the NCEI and NDBC website archives, this step converts the NCEI netCDF pressure units to match the NDBC units (Pa to hPa), and converts the air, water and dew point temperatures from Kelvin to degree Celsius to match NDBC data. This step also performs outlier QA/QC, where it removes zero (‘0‘) wind gust values when no wind speed values are present; direction values greater than 360 °; obvious variable outliers; and duplicated netCDF data points that are ~5–10 seconds apart. To handle the erroneous netCDF spectral frequency data, the code advances through the spectral data and matches the available spectral frequency data to the appropriate 38 frequencies (old wave sensors) or 47 frequencies (new wave sensors).
  3. Step 3: **Verify metadata**. This step is applied solely to the NCEI netCDF data files to validate the netCDF metadata with NDBC-sourced, buoy specific metadata spreadsheets. These metadata spreadsheets were constructed from the NDBC database and original NDBC service technician log books, and provide accurate station and sensor information. Scripts verify or insert missing hull type, payload and mooring type; and verify or insert missing instrument processing systems (for wave data only), instrumentation names and sensor deployment heights. If none are available, metadata fields are augmented with pre-set hull-specific instrumentation specifications that were sourced from online references (for hull-specific instrumentation specifications, please see the USACE QCC Measurement Archive SOP).
  4. Step 4: **Compare, geographically QA/QC and attach metadata**. *Compare*: Although these data originate from the same sensor, storage protocols resulted in different time stamps for each within their various archives. This step compares the NDBC and NCEI sourced data by matching the datasets by nearest date and time (to the minute), after which geographical data are appended to the NDBC datasets.
    As the NDBC data is manually QA/QC’d and does not contain data collected during buoy maintenance operations, these data were considered as a date/time reference to quality control the fixed positions of the pre-2011 netCDF datasets. In other words, if data were present within the NCEI dataset, but not within the NDBC dataset, those NCEI data records were removed.
    Of interest are the datasets within the NCEI netCDF files that pre-date any data published on the NDBC website. These data are likely from sensor and processing tests conducted during deployments that were intentionally not released to the public. These early data are included in the USACE QCC Measurement Archive but have quality control (QC) flags that rate them as unreliable. For more information on these earlier datasets, please reference the technical note on utilizing NDBC data, ERDC/CHL CHETN-I-100^10^.
    *Geographically QA/QC*: Each dataset is filtered to remove GPS positions and associated data that are not within a one (1) degree radius (~60 nautical miles) of the NDBC station watch circles (the surface area through which a buoy can travel while tethered to specific location by a mooring). This radius allows for fluctuations in NDBC deployment locations over the decades, as tests showed that radii of less than one degree significantly removed viable data (see Fig. 2 in the Technical Validation section). Users may wish to further filter their specific datasets to remove additional data points that are outside their target deployment locations; a task now easily achievable with the fully-described, verified metadata included within this USACE QCC Measurement Archive^13^.
    Two methods are used to geographically QA/QC these data: 1) a sorted table of value occurrences to find the most common latitude and longitude positions (using the assumption that the buoy held its correct station for the majority of its life cycle); 2) a manual confirmation and insertion of the primary station locations that were sourced from NDBC buoy specific metadata spreadsheets. This manual step was relevant for buoys that did not consistently hold their stations due to high vandalism rates or strong currents.
    *Assign metadata*: Once the data are geographically QA/QC’d, this step assigns verified metadata (from step 3) to the NDBC stdmet datasets as follows. Station-specific hull type, water depth, payload and mooring type are appended to the NDBC stdmet datasets from the NDBC-sourced, buoy specific metadata spreadsheets. These NDBC Buoy Metadata Spreadsheets and the verified NCEI netCDF metadata are then used to assign the correct primary or secondary sensor designation, which includes metadata such as instrument processing systems (for waves) and instrumentation information (names, deployment heights etc.), to the NDBC stdmet datasets by matching the time paired NDBC variable values with the exact NCEI values.
  5. Step 5: **Create best dataset**. This step selects a combination of the geographically QA/QC datasets that were created in step 4 above. These best available, self-describing datasets (Fig. 1) include:
  NDBC website wind direction, wind speed, wind gust, air pressure at sea level, air temperature, sea surface temperature, significant wave height, dominant and peak periods, mean wave direction, spectral c_11_, alpha_1_, alpha_2_, r_1_, r_2_, with their now fully-described, verified metadata.
  NCEI netCDF spectral ${c}_{11}^{m}$. These data are retained within the USACE QCC Measurement Archive to allow for bulk wave parameter re-calculations without the influences of NDBC shore-side processing protocols.
  Verified station metadata obtained from the NDBC Buoy Metadata Spreadsheets.
  NCEI netCDF data for the above variables that pre-date the NDBC datasets (where applicable).
  6. Step 6: **Create netCDF data files**. This step creates monthly netCDF NDBC data files that collate all of the best available data variables that were selected in step 5 above. For easy access by the USACE and user community, these month-long netCDF data files are stored on the USACE CHL Data Server and are updated annually. A static copy of the historical data (1970–2021) is located within the USACE Knowledge Core Library Datasets^13^.

## Data Records

The static USACE QCC Measurement Archive is stored within the USACE Knowledge Core Library Datasets^13^ (10.21079/11681/43121). The archive includes 141 NDBC stations that have collected data from 1970 through 2021 (where available). Measurement data are stored in monthly netCDF files. For months where all data were available, these monthly, self-describing netCDF files contain 13 station variables and metadata fields, 41 wave data and metadata fields (either 47 or 38 spectral frequency bands), and 28 meteorological data and metadata fields. Metadata are fully described within the variable attributes, including flag number descriptions and metadata source references. All netCDF files contain global attributes that detail general NDBC collection information, World Meteorological Organization (WMO) ID’s and other NDBC station-specific metadata. All netCDF files are Climate and Forecast (CF) compliant with discoverable standard names, and standard missing data fill values of −999.99.

Station metadata variables include time, depth, depthFlag, hull, hullFlag, latitude, latitudeFlag, longitude, longitudeFlag, mooring, mooringFlag, payload, payloadFlag

Wave data and metadata include:

- waveHs (with associated data Flag, Metadata, MetadataFlag)
- waveTm (data Flag, Metadata, MetadataFlag)
- waveTp (data Flag, Metadata, MetadataFlag)
- meanWaveDirection (data Flag, Metadata, MetadataFlag)
- waveFrequencies_47
- waveEnergyDensity_47Frequencies (data Flag, Metadata, MetadataFlag)
- waveEnergyDensityUncorrected_47Frequencies (data Flag, Metadata, MetadataFlag)
- waveAlpha1_47Frequencies (data Flag, Metadata, MetadataFlag)
- waveAlpha2_47Frequencies (data Flag, Metadata, MetadataFlag)
- waveR1_47Frequencies (data Flag, Metadata, MetadataFlag)
- waveR2_47Frequencies (data Flag, Metadata, MetadataFlag)
- waveFrequencies_38
- waveEnergyDensity_38Frequencies (data Flag, Metadata, MetadataFlag)
- waveEnergyDensityUncorrected_38Frequencies (data Flag, Metadata, MetadataFlag)
- waveAlpha1_38Frequencies (data Flag, Metadata, MetadataFlag)
- waveAlpha2_38Frequencies (data Flag, Metadata, MetadataFlag)
- waveR1_38Frequencies (data Flag, Metadata, MetadataFlag)
- waveR2_38Frequencies (data Flag, Metadata, MetadataFlag)

Meteorological data and metadata include:

- windDirection (data Flag, Metadata, MetadataFlag)
- windGust (data Flag, Metadata, MetadataFlag)
- windSpeed (data Flag, Metadata, MetadataFlag)
- surfaceAirPressure (data Flag, Metadata, MetadataFlag)
- surfaceAirTemperature (data Flag, Metadata, MetadataFlag)
- surfaceDewPointTemperature (data Flag, Metadata, MetadataFlag)
- surfaceSeaTemperature (data Flag, Metadata, MetadataFlag)

An active USACE QCC Measurement Archive is located within a buoy section on the CHL Data server and is updated annually.

## Technical Validation

Most data users assume the accuracy of the quality controlled NDBC datasets. Few confirm the geographical position of the data prior to use and rely on outlier checks to identify bad data. However, NDBC only posts the most recent deployment location on their website for each moored buoy site, and does not list historical deployment positions. While NCEI netCDF files do contain hourly GPS data (where available), these datasets also include erroneous wave data that were collected while the buoys are adrift and data that were flagged, but not removed during automated NDBC QA/QC procedures. These wave data are inaccurate as NDBC moored buoy wave data processing algorithms^12^ (e.g. hull response amplitude operators) are not designed to resolve for buoy pitch, roll and heave estimates while untethered to the sea floor, a fundamental component in estimating wave height.

Figure 2 showcases examples of geographical position importance when evaluating NDBC data accuracy. Green track lines and points within the maps and time series plots encapsulate data within an acceptable distance of the deployment location, with red track lines and points highlighting those outside the moored buoy watch circle. Importantly, the time series plots in Fig. 2 showcase how the significant wave height (*H*_*s*_) data reported while the buoy was adrift (red points within the time series data) appears within reasonable ranges and are not easily identifiable as suspect. Hence a liberal geographical limit was placed on the USACE QCC Measurement Archive that removed data exceeding a one degree radius (~ 60 nautical miles) of the median geographical position of the station. This intentionally substantial buffer (~120 nautical miles in total) allows for fluctuating NDBC deployment locations over the decades, as highlighted by the four sites of high density green data points within Station 41001’s GPS positions (Fig. 2, bottom right map). As the USACE QCC Measurement Archive contains fully verified geographical positions for each data point, users may now easily refine these individual stations for their specific time period and deployment location of interest.

**Fig. 3:**
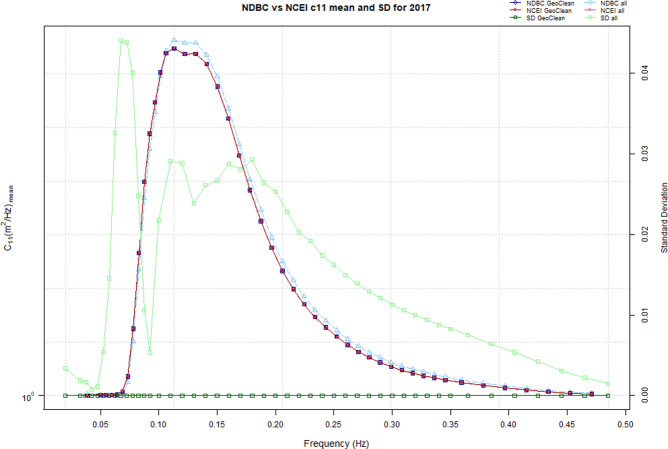
Mean spectral wave energy density (*c*_11_) from NDBC and NCEI data sources for NDBC station 44014 for the year 2017. The geographically cleaned (*GeoClean*) post-QA/QC *c*_11_ are represented by dark blue squares for the NDBC data source, and red squares for the NCEI data source. The pre-QA/QC *all c*_11_ data are represented by light blue triangles for the NDBC data source, and pink triangles for the NCEI data source (where the pink triangles are overshadowed by the post-QA/QC *GeoClean* NDBC blue squares). Measured on the right axis, standard deviations are represented by light green triangles for the pre-QA/QC *all* NDBC and NCEI standard deviations, and dark green squares for the post-QA/QC *GeoClean* NDBC and NCEI standard deviations.

Reviewing the USACE QCC Measurement Archive on a wave spectral level, Fig. 3 shows mean wave energy density (*c*_11_) across the spectral frequency from the NDBC and NCEI data sources for NDBC station 44014 during 2017. The pre-QA/QC *all c*_11_ data are represented by light blue triangles for the NDBC data source, and pink triangles for the NCEI data source (note that the pink triangles are overshadowed by the post-QA/QC *c*_11_ dark blue squares in Fig. 3). These non-QC’d datasets show very little agreement in Fig. 3, which clearly highlights the errors in the NCEI netCDF wave spectral data as these spectral data are duplicates of time-paired data from the same, unique sensor.

After the removal of the erroneous geographical data and outliers, a review of the geographically cleaned (*GeoClean*) post-QA/QC *c*_11_ data from the NDBC (dark blue squares) and the NCEI data sources (red squares) show agreement in that they overlay each other across the spectral frequencies. Additionally, standard deviations between the pre-QA/QC (light green triangles) and post-QA/QC (dark green squares) NDBC and NCEI data show a vast improvement after geographical and outlier checks with the post-QA/QC *c*_11_ standard deviations remaining at unity (zero in this case).

In summary, these typical example evaluations showcase the validity of this USACE QCC Measurement Archive process on the quality of the NDBC meteorological and oceanographic data. Note that this methodology only removes known errors and does not verify data quality from an alternate source. However, this validation and verification work allows for confidence in the NDBC data sourced from the USACE QCC Measurement Archive. Ultimately, this self-describing, consistent USACE QCC Measurement Archive provides measurement data with verified metadata for the accurate evaluation of historical U.S. coastal conditions, which are essential for USACE risk assessment studies, coastal flooding, wave applications, and coastal engineering efforts.

## Usage Notes

For code reviews, users should reference the USACE QCC Measurement Archive SOP document that is stored on the Archive GitHub (https://github.com/CandiceH-CHL/USACE_QCC_measurement_archive.git). This document details the USACE QCC Measurement Archive R code suite that was developed during this work. This R code suite is fully open access and is version controlled. If users plan to reproduce this study, please note that steps two to five in phase one (processing the 1970–2020 historical NDBC data) utilized ~400k high performance computing cpu hours.

Users developing their own quality controlled NDBC Measurement Archive should reference the technical note on utilizing NDBC data, ERDC/CHL CHETN-I-100^10^, which fully details the NDBC website and NCEI stored NDBC netCDF data idiosyncrasies that were discovered during this process. For example, different netCDF file extractions codes are required to handle the multiple NCEI netCDF storage formats. NDBC is in the process of redesigning their present netCDF file format to be more user friendly, but do not plan to edit their archived data. This methodology is structured to handle future NDBC netCDF format changes with minimal code edits.

Additionally, the NCEI netCDF archive routinely contains monthly data that are separated into multiple files with non-sequential suffixes (D1 - D_*n*_). These multiple monthly files indicate that the station was serviced during that month and the data separated to capture the change in instrumentation in the netCDF file metadata. Another note is the similar variable nomenclature that is employed within the NCEI NetCDF files that introduce duplicate data rows if not accounted for, for example: extracting the GPS variable, ‘lon’, from the netCDF files will also capture ‘solar_radiation_sensor_1_ longwave_radiation’ data where available. Please see Appendix E in the technical note on utilizing NDBC data, ERDC/CHL CHETN-I-100^10^, for a list of these similar variable names.

Data archive idiosyncrasies include the use of various column header nomenclature within the historical NDBC website over the decades (please see Appendix D: ERDC/CHL CHETN-I-100^10^ for these changes). Station-specific idiosyncrasies also occur, such as the NDBC website data for station 41009 that has large periods of duplicate NDBC hourly records with no minute information (required manual insertion of minute data as these rows were determined to be unique). The NCEI netCDF data files had similar inaccurate records: for example, the September 2012 netCDF data file for station 41009 contains 2334 10-second continuous wind data fields, 2334 QC flag fields, but only 777 release flag fields. The netCDF files also contain spurious variable place holders that hold no data.

Additionally, while the NDBC website data contain the two sets of wave spectral frequency bands that represent the historically deployed wave instrumentation, the NCEI stored NDBC netCDF files contain multiple sets of wave spectral frequency bands that do not match the NDBC data (please see Appendix F: ERDC/CHL CHETN-I-100^10^ for a list of these erroneous frequencies). Data encased within these non-conforming frequencies bands were treated carefully to only extract the real spectral estimates from amongst the inaccurate wave frequency bands and values.

Finally, data of dubious quality require identification and removal via date/time comparisons with the manually QA/QC’d data, geographical QA/QC and metadata verification. These checks include obvious outlier and erroneous data corrections, including the removal of directional values greater than 360 °; wind gust data that were present when wind speed values were not available; and duplicated netCDF data points that are ~5–10 seconds apart.

## Data Availability

The R source code used to process these data is open access and described in detail in The Comprehensive R Archive Network (https://cran.r-project.org/). The USACE QCC Measurement Archive R code suite developed during this work is open access and is fully described in a USACE QCC Measurement Archive SOP document (https://github.com/CandiceH-CHL/USACE_QCC_measurement_archive.git).
